# Harnessing oncolytic viruses to overcome immunosuppression in breast cancer: from mechanisms to clinical translation

**DOI:** 10.3389/fimmu.2025.1687190

**Published:** 2025-09-10

**Authors:** Kong Xianshu, Liu Zhonghua, Dong Junyu, Peng Qing, Zhang Li, Zhang Feiyue, Su Xuqing

**Affiliations:** ^1^ Department of Breast Surgery, Honghezhou Third People’s Hospital, Honghezhou Cancer Hospital, Gejiu, China; ^2^ Charité-Universitätsmedizin Humboldt University, Berlin, Germany; ^3^ Department of Oncology, the Fifth Affiliated Hospital of Kunming Medical University, Gejiu, China; ^4^ Department of Oncology, the Sixth Affiliated Hospital of Kunming Medical University, YuXi, China

**Keywords:** breast cancer, oncolytic virus, tumor microenvironment, cancer vaccines, combination therapy

## Abstract

Oncolytic viruses (OVs) possess dual advantages in cancer immunotherapy: they selectively replicate within and lyse tumor cells while simultaneously releasing tumor-associated antigens to recruit and activate immune cells within the local tumor microenvironment (TME), thereby inducing robust and sustained antitumor immunity. Furthermore, these viruses can serve as tumor-targeting vectors for immunomodulation and synergize with other immunotherapeutic approaches. As such, oncolytic virotherapy holds significant potential to overcome the low response rates of breast cancer to existing immunotherapies and expand the therapeutic arsenal. This review systematically elucidates the application and mechanisms of this emerging immunotherapy in addressing the challenges of conventional breast cancer treatments. It also discusses engineering strategies to enhance antitumor immunity, highlights recent preclinical and clinical studies on rational combinations of OVs with other therapies, and outlines current challenges and future prospects.

## Introduction

Breast cancer remains a leading cause of cancer-related mortality among women (1), Oncolytic virotherapy (OV), which combines the selective infection and destruction of cancer cells with the induction of adaptive immune responses against tumors, has emerged as a promising frontier in the fight against various cancers, including breast cancer (2–4). Additionally, the integration of OVs with other breast cancer treatments to leverage the strengths of each modality has garnered significant attention as a strategy to address tumor heterogeneity (5–7).

## Mechanisms of oncolytic viruses in breast cancer

**Figure 1:**
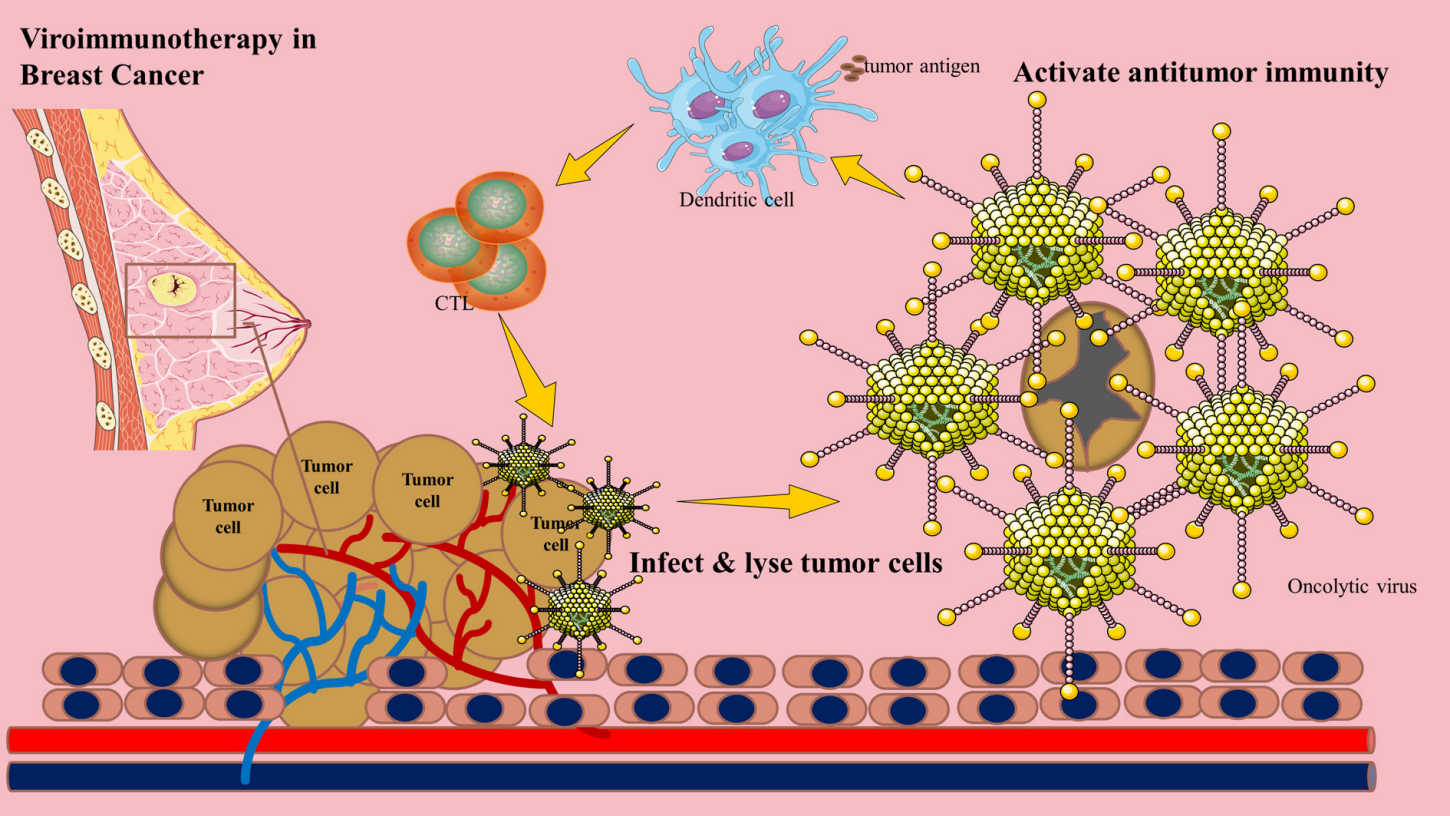
Primary mechanisms of oncolytic virotherapy in breast cancer.

Oncolytic viruses are naturally occurring or genetically engineered immunotherapeutic agents that preferentially replicate in tumors, promoting immunogenic cell death. For instance, the oncolytic mumps virus exhibits potent cytotoxic activity against breast cancer xenografts, and oncolytic peptides demonstrate remarkable antimetastatic properties (8–10). Recombinant OVs have also shown efficacy in triple-negative breast cancer (TNBC) mouse models, a highly aggressive subtype with limited treatment options (11). Beyond direct tumor lysis, OVs primarily exert antitumor effects by activating immune responses (12) **Figure 1**.

The immunosuppressive tumor microenvironment (TME) is a major barrier to cancer immunotherapy (13). By recruiting inhibitory immune cells and upregulating immune checkpoint ligands, the TME fosters immunosuppression and restricts antitumor immunity, facilitating tumor progression and metastasis. OVs remodel the TME by inducing immunogenic cell death, disrupting the dense tumor stroma, and triggering the release of danger signals and tumor-associated antigens. This process attracts antigen-presenting cells, activates and expands lymphocyte populations, and enhances their infiltration into the tumor bed, transforming the TME from an immunologically “cold” to a “hot” state (14–17). T lymphocytes play a pivotal role in this transition.

Insufficient T cell activation limits the efficacy of immunotherapies. The oncolytic parapoxvirus ORFV and its derivatives induce pyroptosis in breast cancer cells and increase intratumoral cytotoxic T lymphocyte (CTL) populations (18, 19). Similarly, the oncolytic vaccinia virus CF33-hNIS-ΔF14.5 enhances CD8+ T cell infiltration in TNBC models (20). A virus-like nanoplatform (PolyIC@ZIF-8) degrades in the acidic TME, releasing PolyIC to induce apoptosis and promote T cell recruitment and activation in an antigen-dependent manner (21). Dendritic cell (DC) activation is also critical for OV-mediated antitumor immunity (22). A GFP-transgenic Newcastle disease virus (NDV-GFP) matures monocyte-derived DCs, priming antigen-specific T cell responses against breast cancer cells (23). Notably, combining high-dose vitamin C with oncolytic adenoviruses (oAds) amplifies T cell activation (24).

Natural killer (NK) cells also contribute to antitumor immunity. An oncolytic vesicular stomatitis virus (VSV)-based vaccine enhances NK cell activity and improves TNBC outcomes (25). OVs engineered to express CCL5 recruit NK cells to tumor sites, synergizing with NK cell-based therapies (26). Moreover, tumor-tropic NK cells can serve as carriers for systemic OV delivery (27) Combining NKT cell immunotherapy with engineered OVs further enhances tumor targeting (28).

## Challenges and optimization strategies for oncolytic virotherapy in breast cancer

### Limited tumor targeting after systemic administration

Enhancing OV specificity for breast cancer cells is a key research focus. For HER2-positive breast cancer, the HSV-based OV R-LM249 selectively infects and kills HER2-overexpressing cells (29) For HER2-negative tumors, mesothelin (MSLN) is a promising target (30). The recombinant measles virus rMV-SLAMblind suppresses Nectin-4-positive TNBC cells (31). Bispecific T cell engagers (BiTEs) can redirect T cells to tumor antigens, and OV-BiTE combinations represent a novel targeting strategy (32). For instance, a PD-L1-targeting BiTE-armed oHSV-1 selectively kills PD-L1+ tumor cells and macrophages while sparing T cells (33). Intratumoral OV delivery also minimizes off-target toxicity (34).

### Host antiviral immune responses

Neutralizing antibodies pose a major challenge to systemic OV delivery. Strategies to evade or repurpose these antibodies are under investigation (35, 36). Antibody retargeting improves intratumoral adenovirus efficacy (37). Magnetic nanoparticles conjugated to HSV1716 shield the virus from neutralizing antibodies and enable magnetic tumor targeting (38). Mesenchymal stem cells (MSCs) serve as effective OV carriers, enhancing tumor delivery and infiltration (39, 40). A liposome-encapsulated NDV expressing MIP-3α stimulates antitumor immunity and inhibits angiogenesis (41). Exosome-based delivery systems also show promise in TNBC (42).

### Engineering multifunctional OVs

Genetic modifications enhance OV specificity and potency. A miR-145/143-modified coxsackievirus B3 (miR-CVB3-1.1) selectively lyses breast cancer cells (43). Combining miR-CVB3 with CpG-melittin suppresses primary and metastatic tumor growth (44). The oncolytic adenovirus AdSVP-lncRNAi9 silences oncogenic miRNAs to inhibit TNBC proliferation and migration (45).

Immunomodulatory OVs amplify antitumor immunity. IL-21- or IL-23-armed vaccinia viruses induce potent immune responses (46, 47). A TK/N1L-deleted vaccinia virus (VVΔTKΔN1L) prevents postoperative recurrence and metastasis (48) TGF-β inhibition synergizes with OVs (49)., and deleting immune evasion genes enhances efficacy (50). Besides, OVs expressing PD-1/IL-12 remodel the immunosuppressive TME (51).

Immune checkpoint-armed OVs combine virotherapy with checkpoint blockade. An adenovirus suppressing PD-L1 improves checkpoint inhibitor safety and efficacy (52). A TIGIT-targeting scFv-armed vaccinia virus (VV-scFv-TIGIT) synergizes with PD-1 blockade (53). Another engineered VV-α-TIGIT enhances T cell recruitment and activation (54).

## Combination therapies with oncolytic viruses

OVs are potent modulators of the TME, and their combination with immune checkpoint inhibitors (ICIs), CAR-T cells, or other immunotherapies represents a highly promising strategy (55–58). For instance, the combination of reovirus and CD3-bispecific antibodies enhances interferon-mediated responses and promotes T cell infiltration, leading to tumor regression in HER2+ breast cancer models (59). Additionally, genome-wide CRISPR-Cas9 screening has identified PARP1 as a key cellular factor that restricts viral replication; accordingly, PARP inhibition sensitizes TNBC to OV-ICI combination therapy (60) Neoadjuvant OV treatment has also been shown to improve surgical outcomes and reduce recurrence rates (61). It is critical to optimize dosing and timing in these combinational approaches, as the synergistic effects between OVs and ICIs are both dose- and schedule-dependent (62, 63) Therefore, establishing optimal dosing regimens and treatment sequences is paramount for the rational design of clinical trials investigating OV-based combination immunotherapies.

OVs also synergize with chemotherapy, radiotherapy, and targeted therapies (64). **Table 1** Stereotactic body radiotherapy (SBRT) enhances OV-induced immunogenic cell death (65). Kinase inhibitors, such as BRAF inhibitors, improve OV efficacy (66, 67). Epigenetic modifiers like entinostat augment OV-IL-15 superagonist combinations (68, 69).

**Table 1:** Pre-clinical research of oncolytic virotherapy in breast cancer.

Oncolytic virus	Combined agent	Mechanism of action	Key findings	Ref
Oncolytic alphavirus M1	Doxorubicin	Doxorubicin enhances viral replication and necroptosis	Suppresses TNBC growth *in vivo*	(70)
Reovirus	Doxorubicin conjugate	Reo-dox promotes innate immune activation and DNA damage	Reduces primary and metastatic TNBC burden	(71)
Talimogene laherparepvec (T-VEC)	NAC	T-VEC + neoadjuvant chemotherapy (NAC) for TNBC (Phase I trial)	55% complete response rate, manageable toxicity	(72)
T-VEC	Paclitaxel + NAC	Intratumoral T-VEC + chemotherapy (Phase II trial)	Increases RCB0–1 rates in TNBC	(73)
VG161 (oHSV-1)	Paclitaxel	Induces proinflammatory TME changes	Inhibits breast cancer growth and lung metastasis	(74)
TG6002 (VV)	5-Fluorouracil	Converts 5-FC to 5-FU, inducing tumor lysis	Effective in canine mammary tumors	(75)
Measles virus (MV)	Baicalein (BAI) + Cinnamaldehyde (CIN)	BAI/CIN sensitize tumor cells to MV	Enhances tumor cell killing。	(76)
VSVΔ51	T-DM1	VSVΔ51 overcomes T-DM1 resistance in HER2+ cells	Improves survival in HER2+ xenografts	(77)
T-VEC	Atezolizumab	T-VEC + anti-PD-L1 for TNBC (Phase Ib trial)	Limited antitumor activity observed	(78)

T-DM1 (Kadcyla^®^), an antibody-drug conjugate; atezolizumab, an immune checkpoint inhibitor.

## Development of potent oncolytic virus cancer vaccines

The development of robust oncolytic virus (OV)-based cancer vaccines relies on the rational design of tumor-selective viruses and the strategic exploitation of their immunostimulatory properties. Utilizing OVs as an adjuvant platform for therapeutic cancer vaccines is particularly attractive for personalized immunotherapy targeting patient-specific neoantigens (79, 80). High-throughput sequencing technologies can be leveraged to optimize viral design, modulate immune responses, and identify predictive biomarkers of clinical efficacy (81). Furthermore, direct imaging and automated analysis using tumor-on-chip systems have elucidated the cooperative antitumor activity between immune cells and oncolytic vaccinia virus, providing novel insights into the mechanisms of action of oncolytic vaccines (82).

## Discussion

Oncolytic virotherapy represents a novel multimodal approach bridging virology, oncology, and immunology. While preclinical and clinical studies validate the antitumor effects of several OVs, their clinical translation faces challenges, including immune and physical barriers that limit intratumoral delivery, replication, and spread. Beyond improving OV bioavailability and efficacy, developing platforms that synergize with existing therapies is crucial. A deeper understanding of host-virus interactions, particularly in metabolically relevant models, will help bridge the gap between bench and bedside.

## References

[1] Siegel RL Kratzer TB Giaquinto AN Sung H Jemal A. Cancer statistics, 2025. CA Cancer J Clin. (2025) 75:10–45. doi: 10.3322/caac.21871, PMID: 39817679 PMC11745215

[2] Ma XY Hill BD Hoang T Wen F. Virus-inspired strategies for cancer therapy. Semin Cancer Biol. (2022) 86:1143–57. doi: 10.1016/j.semcancer.2021.06.021, PMID: 34182141 PMC8710185

[3] Javanbakht M Tahmasebzadeh S Cegolon L Gholami N Kashaki M Nikoueinejad H. Oncolytic viruses: A novel treatment strategy for breast cancer. Genes Dis. (2023) 10:430–46. doi: 10.1016/j.gendis.2021.11.011, PMID: 37223527 PMC10201557

[4] Li Z Feiyue Z Gaofeng L Haifeng L. Lung cancer and oncolytic virotherapy–enemy’s enemy. Transl Oncol. (2023) 27:101563. doi: 10.1016/j.tranon.2022.101563, PMID: 36244134 PMC9561464

[5] Bahreyni A Mohamud Y Luo H. Oncolytic virus-based combination therapy in breast cancer. Cancer Lett. (2024) 585:216634. doi: 10.1016/j.canlet.2024.216634, PMID: 38309616

[6] Lin W Zhao Y Zhong L. Current strategies of virotherapy in clinical trials for cancer treatment. J Med Virol. (2021) 93:4668–92. doi: 10.1002/jmv.26947, PMID: 33738818

[7] de Graaf JF Huberts M Fouchier RAM van den Hoogen BG. Determinants of the efficacy of viro-immunotherapy: A review. Cytokine Growth Factor Rev. (2020) 56:124–32. doi: 10.1016/j.cytogfr.2020.07.001, PMID: 32919831

[8] Hartley A Kavishwar G Salvato I Marchini A. A roadmap for the success of oncolytic parvovirus-based anticancer therapies. Annu Rev Virol. (2020) 7:537–57. doi: 10.1146/annurev-virology-012220-023606, PMID: 32600158

[9] Behrens MD Stiles RJ Pike GM Sikkink LA Zhuang Y Yu J. Oncolytic Urabe mumps virus: A promising virotherapy for triple-negative breast cancer. Mol Ther Oncolytics. (2022) 27:239–55. doi: 10.1016/j.omto.2022.11.002, PMID: 36458203 PMC9703006

[10] Tang T Huang X Zhang G Liang T. Oncolytic immunotherapy: multiple mechanisms of oncolytic peptides to confer anticancer immunity. J Immunother Cancer. (2022) 10:e005065. doi: 10.1136/jitc-2022-005065, PMID: 35851309 PMC9295653

[11] Thomas RJ Bartee MY Valenzuela-Cardenas M Bartee E. Oncolytic myxoma virus is effective in murine models of triple negative breast cancer despite poor rates of infection. Mol Ther Oncolytics. (2023) 30:316–9. doi: 10.1016/j.omto.2023.08.014, PMID: 37732297 PMC10507476

[12] Feola S Russo S Ylösmäki E Cerullo V. Oncolytic ImmunoViroTherapy: A long history of crosstalk between viruses and immune system for cancer treatment. Pharmacol Ther. (2022) 236:108103. doi: 10.1016/j.pharmthera.2021.108103, PMID: 34954301

[13] Hemminki O Dos Santos JM Hemminki A. Oncolytic viruses for cancer immunotherapy. J Hematol Oncol. (2020) 13:84. doi: 10.1186/s13045-020-00922-1, PMID: 32600470 PMC7325106

[14] Jamieson TR Poutou J Ilkow CS. Redirecting oncolytic viruses: Engineering opportunists to take control of the tumour microenvironment. Cytokine Growth Factor Rev. (2020) 56:102–14. doi: 10.1016/j.cytogfr.2020.07.004, PMID: 32958389

[15] DePeaux K Delgoffe GM. Metabolic barriers to cancer immunotherapy. Nat Rev Immunol. (2021) 21:785–97. doi: 10.1038/s41577-021-00541-y, PMID: 33927375 PMC8553800

[16] Zhang B Cheng P. Improving antitumor efficacy via combinatorial regimens of oncolytic virotherapy. Mol Cancer. (2020) 19:158. doi: 10.1186/s12943-020-01275-6, PMID: 33172438 PMC7656670

[17] Pol JG Workenhe ST Konda P Gujar S Kroemer G. Cytokines in oncolytic virotherapy. Cytokine Growth Factor Rev. (2020) 56:4–27. doi: 10.1016/j.cytogfr.2020.10.007, PMID: 33183957

[18] Su W Qiu W Li SJ Wang S Xie J Yang QC. A dual-responsive STAT3 inhibitor nanoprodrug combined with oncolytic virus elicits synergistic antitumor immune responses by igniting pyroptosis. Adv Mater. (2023) 35:e2209379. doi: 10.1002/adma.202209379, PMID: 36545949

[19] Lin J Sun S Zhao K Gao F Wang R Li Q. Oncolytic Parapoxvirus induces Gasdermin E-mediated pyroptosis and activates antitumor immunity. Nat Commun. (2023) 14:224. doi: 10.1038/s41467-023-35917-2, PMID: 36641456 PMC9840172

[20] Chaurasiya S Yang A Kang S Lu J Kim SI Park AK. Oncolytic poxvirus CF33-hNIS-ΔF14.5 favorably modulates tumor immune microenvironment and works synergistically with anti-PD-L1 antibody in a triple-negative breast cancer model. Oncoimmunology. (2020) 9:1729300. doi: 10.1080/2162402X.2020.1729300, PMID: 32158622 PMC7051185

[21] Wu F Li Y Meng Y Cai X Shi J Li J. An ion-enhanced oncolytic virus-like nanoparticle for tumor immunotherapy. Angew Chem Int Ed Engl. (2022) 61:e202210487., PMID: 36117387 10.1002/anie.202210487

[22] Wang W Liu S Dai P Yang N Wang Y Giese RA. Elucidating mechanisms of antitumor immunity mediated by live oncolytic vaccinia and heat-inactivated vaccinia. J Immunother Cancer. (2021) 9:e002569. doi: 10.1136/jitc-2021-002569, PMID: 34593618 PMC8487208

[23] Xu Q Rangaswamy US Wang W Robbins SH Harper J Jin H. Evaluation of Newcastle disease virus mediated dendritic cell activation and cross-priming tumor-specific immune responses ex vivo. Int J Cancer. (2020) 146:531–41. doi: 10.1002/ijc.32694, PMID: 31584185

[24] Ma J Zhang C Shi G Yue D Shu Y Hu S. High-dose VitC plus oncolytic adenoviruses enhance immunogenic tumor cell death and reprogram tumor immune microenvironment. Mol Ther. (2022) 30:644–61. doi: 10.1016/j.ymthe.2021.09.015, PMID: 34547462 PMC8821933

[25] Niavarani SR Lawson C Boudaud M Simard C Tai LH. Oncolytic vesicular stomatitis virus-based cellular vaccine improves triple-negative breast cancer outcome by enhancing natural killer and CD8(+) T-cell functionality. J Immunother Cancer. (2020) 8:e000465. doi: 10.1136/jitc-2019-000465, PMID: 32179632 PMC7073779

[26] Li F Sheng Y Hou W Sampath P Byrd D Thorne S. CCL5-armed oncolytic virus augments CCR5-engineered NK cell infiltration and antitumor efficiency. J Immunother Cancer. (2020) 8:e000131. doi: 10.1136/jitc-2019-000131, PMID: 32098828 PMC7057442

[27] Ding L Gao Q Xu Z Cai L Chen S Zhang X. An inter-supplementary biohybrid system based on natural killer cells for the combinational immunotherapy and virotherapy of cancer. Adv Sci (Weinh). (2022) 9:e2103470. doi: 10.1002/advs.202103470, PMID: 34747156 PMC8805568

[28] Nelson A McMullen N Gebremeskel S De Antueno R Mackenzie D Duncan R. Fusogenic vesicular stomatitis virus combined with natural killer T cell immunotherapy controls metastatic breast cancer. Breast Cancer Res. (2024) 26:78. doi: 10.1186/s13058-024-01818-5, PMID: 38750591 PMC11094881

[29] Nanni P Gatta V Menotti L De Giovanni C Ianzano M Palladini A. Preclinical therapy of disseminated HER-2^+^ ovarian and breast carcinomas with a HER-2-retargeted oncolytic herpesvirus. PLoS Pathog. (2013) 9:e1003155. doi: 10.1371/journal.ppat.1003155, PMID: 23382683 PMC3561254

[30] Froechlich G Gentile C Infante L Caiazza C Pagano P Scatigna S. Generation of a novel mesothelin-targeted oncolytic herpes virus and implemented strategies for manufacturing. Int J Mol Sci. (2021) 22:477. doi: 10.3390/ijms22020477, PMID: 33418877 PMC7825047

[31] Fujiyuki T Amagai Y Shoji K Kuraishi T Sugai A Awano M. Recombinant SLAMblind measles virus is a promising candidate for nectin-4-positive triple negative breast cancer therapy. Mol Ther Oncolytics. (2020) 19:127–35. doi: 10.1016/j.omto.2020.09.007, PMID: 33145396 PMC7585052

[32] Heidbuechel JPW Engeland CE. Oncolytic viruses encoding bispecific T cell engagers: a blueprint for emerging immunovirotherapies. J Hematol Oncol. (2021) 14:63. doi: 10.1186/s13045-021-01075-5, PMID: 33863363 PMC8052795

[33] Khalique H Baugh R Dyer A Scott EM Frost S Larkin S. Oncolytic herpesvirus expressing PD-L1 BiTE for cancer therapy: exploiting tumor immune suppression as an opportunity for targeted immunotherapy. J Immunother Cancer. (2021) 9:e001292. doi: 10.1136/jitc-2020-001292, PMID: 33820820 PMC8026026

[34] Hong WX Haebe S Lee AS Westphalen CB Norton JA Jiang W. Intratumoral immunotherapy for early-stage solid tumors. Clin Cancer Res. (2020) 26:3091–9. doi: 10.1158/1078-0432.CCR-19-3642, PMID: 32071116 PMC7439755

[35] Ma R Li Z Chiocca EA Caligiuri MA Yu J. The emerging field of oncolytic virus-based cancer immunotherapy. Trends Cancer. (2023) 9:122–39. doi: 10.1016/j.trecan.2022.10.003, PMID: 36402738 PMC9877109

[36] Shin DH Nguyen T Ozpolat B Lang F Alonso M Gomez-Manzano C. Current strategies to circumvent the antiviral immunity to optimize cancer virotherapy. J Immunother Cancer. (2021) 9:e002086. doi: 10.1136/jitc-2020-002086, PMID: 33795384 PMC8021759

[37] Niemann J Woller N Brooks J Fleischmann-Mundt B Martin NT Kloos A. Molecular retargeting of antibodies converts immune defense against oncolytic viruses into cancer immunotherapy. Nat Commun. (2019) 10:3236. doi: 10.1038/s41467-019-11137-5, PMID: 31324774 PMC6642145

[38] Howard FHN Al-Janabi H Patel P Cox K Smith E Vadakekolathu J. Nanobugs as drugs: bacterial derived nanomagnets enhance tumor targeting and oncolytic activity of HSV-1 virus. Small. (2022) 18:e2104763. doi: 10.1002/smll.202104763, PMID: 35076148

[39] Moreno R. Mesenchymal stem cells and oncolytic viruses: joining forces against cancer. J Immunother Cancer. (2021) 9:e001684. doi: 10.1136/jitc-2020-001684, PMID: 33558278 PMC7871674

[40] Seyed-Khorrami SM Soleimanjahi H Soudi S Habibian A. MSCs loaded with oncolytic reovirus: migration and *in vivo* virus delivery potential for evaluating anti-cancer effect in tumor-bearing C57BL/6 mice. Cancer Cell Int. (2021) 21:244. doi: 10.1186/s12935-021-01848-5, PMID: 33933086 PMC8088007

[41] Wang JY Chen H Dai SZ Huang FY Lin YY Wang CC. Immunotherapy combining tumor and endothelium cell lysis with immune enforcement by recombinant MIP-3α Newcastle disease virus in a vessel-targeting liposome enhances antitumor immunity. J Immunother Cancer. (2022) 10:e003950. doi: 10.1136/jitc-2021-003950, PMID: 35256516 PMC8905871

[42] Zhang C Tang S Wang M Li L Li J Wang D. Triple-punch” Strategy exosome-mimetic nanovesicles for triple negative breast cancer therapy. ACS Nano. (2024), 3c10568. doi: 10.1021/acsnano.3c10568, PMID: 38335265

[43] Bahreyni A Liu H Mohamud Y Xue YC Zhang J Luo H. A new miRNA-Modified coxsackievirus B3 inhibits triple negative breast cancer growth with improved safety profile in immunocompetent mice. Cancer Lett. (2022) 548:215849. doi: 10.1016/j.canlet.2022.215849, PMID: 35995138

[44] Bahreyni A Liu H Mohamud Y Xue YC Fan YM Zhang YL. A combination of genetically engineered oncolytic virus and melittin-CpG for cancer viro-chemo-immunotherapy. BMC Med. (2023) 21:193. doi: 10.1186/s12916-023-02901-y, PMID: 37226233 PMC10210435

[45] Ang L Guo L Wang J Huang J Lou X Zhao M. Oncolytic virotherapy armed with an engineered interfering lncRNA exhibits antitumor activity by blocking the epithelial mesenchymal transition in triple-negative breast cancer. Cancer Lett. (2020) 479:42–53. doi: 10.1016/j.canlet.2020.03.012, PMID: 32200038

[46] Chen T Ding X Liao Q Gao N Chen Y Zhao C. IL-21 arming potentiates the anti-tumor activity of an oncolytic vaccinia virus in monotherapy and combination therapy. J Immunother Cancer. (2021) 9:e001647. doi: 10.1136/jitc-2020-001647, PMID: 33504576 PMC7843316

[47] Chen L Chen H Ye J Ge Y Wang H Dai E. Intratumoral expression of interleukin 23 variants using oncolytic vaccinia virus elicit potent antitumor effects on multiple tumor models via tumor microenvironment modulation. Theranostics. (2021) 11:6668–81. doi: 10.7150/thno.56494, PMID: 34093846 PMC8171085

[48] Ahmed J Chard LS Yuan M Wang J Howells A Li Y. A new oncolytic Vacciniavirus augments antitumor immune responses to prevent tumor recurrence and metastasis after surgery. J Immunother Cancer. (2020) 8:e000415., PMID: 32217766 10.1136/jitc-2019-000415 PMC7206973

[49] Groeneveldt C van Hall T van der Burg SH Ten Dijke P van Montfoort N. Immunotherapeutic potential of TGF-β Inhibition and oncolytic viruses. Trends Immunol. (2020) 41:406–20. doi: 10.1016/j.it.2020.03.003, PMID: 32223932

[50] Umer BA Noyce RS Franczak BC Shenouda MM Kelly RG Favis NA. Deciphering the immunomodulatory capacity of oncolytic vaccinia virus to enhance the immune response to breast cancer. Cancer Immunol Res. (2020) 8:618–31. doi: 10.1158/2326-6066.CIR-19-0703, PMID: 32127390

[51] Valenzuela-Cardenas M Gowan C Dryja P Bartee MY Bartee E. TNF blockade enhances the efficacy of myxoma virus-based oncolytic virotherapy. J Immunother Cancer. (2022) 10:e004770. doi: 10.1136/jitc-2022-004770, PMID: 35577502 PMC9114862

[52] Hamdan F Ylösmäki E Chiaro J Giannoula Y Long M Fusciello M. Novel oncolytic adenovirus expressing enhanced cross-hybrid IgGA Fc PD-L1 inhibitor activates multiple immune effector populations leading to enhanced tumor killing *in vitro*, *in vivo* and with patient-derived tumor organoids. J Immunother Cancer. (2021) 9:e003000. doi: 10.1136/jitc-2021-003000, PMID: 34362830 PMC8351494

[53] Zuo S Wei M Xu T Kong L He B Wang S. An engineered oncolytic vaccinia virus encoding a single-chain variable fragment against TIGIT induces effective antitumor immunity and synergizes with PD-1 or LAG-3 blockade. J Immunother Cancer. (2021) 9:e002843. doi: 10.1136/jitc-2021-002843, PMID: 34949694 PMC8705214

[54] Zuo S Wei M He B Chen A Wang S Kong L. Enhanced antitumor efficacy of a novel oncolytic vaccinia virus encoding a fully monoclonal antibody against T-cell immunoglobulin and ITIM domain (TIGIT). EBioMedicine. (2021) 64:103240. doi: 10.1016/j.ebiom.2021.103240, PMID: 33581644 PMC7878184

[55] Zhu Z McGray AJR Jiang W Lu B Kalinski P Guo ZS. Improving cancer immunotherapy by rationally combining oncolytic virus with modulators targeting key signaling pathways. Mol Cancer. (2022) 21:196. doi: 10.1186/s12943-022-01664-z, PMID: 36221123 PMC9554963

[56] Melcher A Harrington K Vile R. Oncolytic virotherapy as immunotherapy. Science. (2021) 374:1325–6. doi: 10.1126/science.abk3436, PMID: 34882456 PMC8961675

[57] Tian Y Xie D Yang L. Engineering strategies to enhance oncolytic viruses in cancer immunotherapy. Signal Transduct Target Ther. (2022) 7:117. doi: 10.1038/s41392-022-00951-x, PMID: 35387984 PMC8987060

[58] Kanaya N Kuroda S Kakiuchi Y Kumon K Tsumura T Hashimoto M. Immune modulation by telomerase-specific oncolytic adenovirus synergistically enhances antitumor efficacy with anti-PD1 antibody. Mol Ther. (2020) 28:794–804. doi: 10.1016/j.ymthe.2020.01.003, PMID: 31991110 PMC7054725

[59] Groeneveldt C Kinderman P van den Wollenberg DJM van den Oever RL Middelburg J Mustafa DAM. Preconditioning of the tumor microenvironment with oncolytic reovirus converts CD3-bispecific antibody treatment into effective immunotherapy. J Immunother Cancer. (2020) 8:e001191. doi: 10.1136/jitc-2020-001191, PMID: 33082167 PMC7577070

[60] Zhong Y Le H Zhang X Dai Y Guo F Ran X. Identification of restrictive molecules involved in oncolytic virotherapy using genome-wide CRISPR screening. J Hematol Oncol. (2024) 17:36. doi: 10.1186/s13045-024-01554-5, PMID: 38783389 PMC11118103

[61] Mullins-Dansereau V Petrazzo G Geoffroy K Béland D Bourgeois-Daigneault MC. Pre-surgical oncolytic virotherapy improves breast cancer outcomes. Oncoimmunology. (2019) 8:e1655363. doi: 10.1080/2162402X.2019.1655363, PMID: 31646102 PMC6791421

[62] Flores EB Aksoy BA Bartee E. Initial dose of oncolytic myxoma virus programs durable antitumor immunity independent of *in vivo* viral replication. J Immunother Cancer. (2020) 8:e000804. doi: 10.1136/jitc-2020-000804, PMID: 32581062 PMC7319776

[63] Nguyen HM Bommareddy PK Silk AW Saha D. Optimal timing of PD-1 blockade in combination with oncolytic virus therapy. Semin Cancer Biol. (2022) 86:971–80. doi: 10.1016/j.semcancer.2021.05.019, PMID: 34033895

[64] Roulstone V Mansfield D Harris RJ Twigger K White C de Bono J. Antiviral antibody responses to systemic administration of an oncolytic RNA virus: the impact of standard concomitant anticancer chemotherapies. J Immunother Cancer. (2021) 9:e002673. doi: 10.1136/jitc-2021-002673, PMID: 34301814 PMC8728387

[65] Chen WY Chen YL Lin HW Chang CF Huang BS Sun WZ. Stereotactic body radiation combined with oncolytic vaccinia virus induces potent anti-tumor effect by triggering tumor cell necroptosis and DAMPs. Cancer Lett. (2021) 523:149–61. doi: 10.1016/j.canlet.2021.09.040, PMID: 34606928

[66] Gilchrist VH Jémus-Gonzalez E Said A Alain T. Kinase inhibitors with viral oncolysis: Unmasking pharmacoviral approaches for cancer therapy. Cytokine Growth Factor Rev. (2020) 56:83–93. doi: 10.1016/j.cytogfr.2020.07.008, PMID: 32690442

[67] Crespo-Rodriguez E Bergerhoff K Bozhanova G Foo S Patin EC Whittock H. Combining BRAF inhibition with oncolytic herpes simplex virus enhances the immune-mediated antitumor therapy of BRAF-mutant thyroid cancer. J Immunother Cancer. (2020) 8:e000698. doi: 10.1136/jitc-2020-000698, PMID: 32759235 PMC7445339

[68] Murphy SA Mapes NJ Jr. Dua D Kaur B. Histone modifiers at the crossroads of oncolytic and oncogenic viruses. Mol Ther. (2022) 30:2153–62. doi: 10.1016/j.ymthe.2022.02.006, PMID: 35143960 PMC9171252

[69] Hicks KC Knudson KM Lee KL Hamilton DH Hodge JW Figg WD. Cooperative immune-mediated mechanisms of the HDAC inhibitor entinostat, an IL15 superagonist, and a cancer vaccine effectively synergize as a novel cancer therapy. Clin Cancer Res. (2020) 26:704–16. doi: 10.1158/1078-0432.CCR-19-0727, PMID: 31645354 PMC8274944

[70] Zhang J Liu Y Tan J Zhang Y Wong CW Lin Z. Necroptotic virotherapy of oncolytic alphavirus M1 cooperated with Doxorubicin displays promising therapeutic efficacy in TNBC. Oncogene. (2021) 40:4783–95. doi: 10.1038/s41388-021-01869-4, PMID: 34155344

[71] Berry JTL Muñoz LE Rodríguez Stewart RM Selvaraj P Mainou BA. Doxorubicin conjugation to reovirus improves oncolytic efficacy in triple-negative breast cancer. Mol Ther Oncolytics. (2020) 18:556–72. doi: 10.1016/j.omto.2020.08.008, PMID: 32995480 PMC7493048

[72] Soliman H Hogue D Han H Mooney B Costa R Lee MC. A phase I trial of talimogene laherparepvec in combination with neoadjuvant chemotherapy for the treatment of nonmetastatic triple-negative breast cancer. Clin Cancer Res. (2021) 27:1012–8. doi: 10.1158/1078-0432.CCR-20-3105, PMID: 33219014

[73] Soliman H Hogue D Han H Mooney B Costa R Lee MC. Oncolytic T-VEC virotherapy plus neoadjuvant chemotherapy in nonmetastatic triple-negative breast cancer: a phase 2 trial. Nat Med. (2023) 29:450–7. doi: 10.1038/s41591-023-02309-4, PMID: 36759673

[74] Deng X Shen Y Yi M Zhang C Zhao B Zhong G. Combination of novel oncolytic herpesvirus with paclitaxel as an efficient strategy for breast cancer therapy. J Med Virol. (2023) 95:e28768. doi: 10.1002/jmv.28768, PMID: 37212336

[75] Béguin J Foloppe J Maurey C Laloy E Hortelano J Nourtier V. Preclinical evaluation of the oncolytic vaccinia virus TG6002 by translational research on canine breast cancer. Mol Ther Oncolytics. (2020) 19:57–66. doi: 10.1016/j.omto.2020.08.020, PMID: 33072863 PMC7533293

[76] Kuo YT Liu CH Wong SH Pan YC Lin LT. Small molecules baicalein and cinnamaldehyde are potentiators of measles virus-induced breast cancer oncolysis. Phytomedicine. (2021) 89:153611. doi: 10.1016/j.phymed.2021.153611, PMID: 34144429

[77] Arulanandam R Taha Z Garcia V Selman M Chen A Varette O. The strategic combination of trastuzumab emtansine with oncolytic rhabdoviruses leads to therapeutic synergy. Commun Biol. (2020) 3:254. doi: 10.1038/s42003-020-0972-7, PMID: 32444806 PMC7244474

[78] Hecht JR Raman SS Chan A Kalinsky K Baurain JF Jimenez MM. Phase Ib study of talimogene laherparepvec in combination with atezolizumab in patients with triple negative breast cancer and colorectal cancer with liver metastases. ESMO Open. (2023) 8:100884. doi: 10.1016/j.esmoop.2023.100884, PMID: 36863095 PMC10163149

[79] Dyer A Frost S Fisher KD Seymour LW. The role of cancer metabolism in defining the success of oncolytic viro-immunotherapy. Cytokine Growth Factor Rev. (2020) 56:115–23. doi: 10.1016/j.cytogfr.2020.07.006, PMID: 32921554

[80] Roy DG Geoffroy K Marguerie M Khan ST Martin NT Kmiecik J. Adjuvant oncolytic virotherapy for personalized anti-cancer vaccination. Nat Commun. (2021) 12:2626. doi: 10.1038/s41467-021-22929-z, PMID: 33976179 PMC8113265

[81] Naik S Russell L. The 13(th) International Oncolytic Virus Conference: Powerful payloads gain clinical momentum. Mol Ther. (2022) 30:1361–3. doi: 10.1016/j.ymthe.2022.03.010, PMID: 35349786 PMC9077475

[82] Mencattini A Lansche C Veith I Erbs P Balloul JM Quemeneur E. Direct imaging and automatic analysis in tumor-on-chip reveal cooperative antitumoral activity of immune cells and oncolytic vaccinia virus. Biosens Bioelectron. (2022) 215:114571. doi: 10.1016/j.bios.2022.114571, PMID: 35932554

